# Current Insights into Migratory Endoparasitism: Deciphering the Biology, Parasitism Mechanisms, and Management Strategies of Key Migratory Endoparasitic Phytonematodes

**DOI:** 10.3390/plants9060671

**Published:** 2020-05-26

**Authors:** Reny Mathew, Charles H. Opperman

**Affiliations:** Department of Entomology and Plant Pathology, North Carolina State University, Raleigh, NC 27695, USA; rmathew2@ncsu.edu

**Keywords:** migratory nematodes, *Radopholus*, *Pratylenchus*, *Ditylenchus*, Bursaphelenchus, plant-parasitic nematodes, parasitism genes

## Abstract

Despite their physiological differences, sedentary and migratory plant-parasitic nematodes (PPNs) share several commonalities. Functional characterization studies of key effectors and their targets identified in sedentary phytonematodes are broadly applied to migratory PPNs, generalizing parasitism mechanisms existing in distinct lifestyles. Despite their economic significance, host–pathogen interaction studies of migratory endoparasitic nematodes are limited; they have received little attention when compared to their sedentary counterparts. Because several migratory PPNs form disease complexes with other plant-pathogens, it is important to understand multiple factors regulating their feeding behavior and lifecycle. Here, we provide current insights into the biology, parasitism mechanism, and management strategies of the four-key migratory endoparasitic PPN genera, namely *Pratylenchus, Radopholus, Ditylenchus*, and *Bursaphelenchus*. Although this review focuses on these four genera, many facets of feeding mechanisms and management are common across all migratory PPNs and hence can be applied across a broad genera of migratory phytonematodes.

## 1. Introduction

Of approximately 27,000 described nematode species, roughly 4100 utilize higher terrestrial plants as a predominant source of nutrition [1]. These plant-parasitic nematodes (PPNs), cause ~$80–$157 billion crop losses annually worldwide [2,3]. The earliest evidence of a nematode identified within a plant is the fossilized eggs, juveniles, and adults of *Palaeonema phyticum* (Poinar, Kerp, and Hass, 2008) in the stem of the terrestrial plant *Aglaophyton major* (Kidson and Lang, 1920) in the Devonian era [4]. This discovery of *P. phyticum* uncovered an ancient and a pivotal point in the timeline of transition of nematodes from free-living to parasites of land plants. Since microbes were found in the stomatal spaces invaded by the nematode, *P. phyticum* was tentatively categorized as a facultative plant-parasite, belonging to clade 1 of the phylum Nematoda (due to the morphological similarities to clade 1 nematodes) [4,5,6]. Feeding on fungi or bacteria or other microbes is also considered to be one of the most important determinants for the evolution of facultative or obligate plant-parasitism in nematodes. This evolution is thought to have occurred independently four times within Nematoda [4,5,7,8]. In the phylogenetic tree outlined by Van Megen et al., these events place PPNs in four of the twelve clades: 1 (Triplonchida), 2 (Dorylaimida), 10 (Aphelenchoididae), and 12 (Tylenchida). A prominent morphologically distinctive feature of all PPNs is a protrusible needle-like apparatus known as the stylet. The stylet, an artifact of convergent evolution (within the above-mentioned four clades) is utilized by PPNs for two main reasons: (1) puncturing the plant cell wall to extract cell nutrients, and (2) in certain nematode species, delivering secretory molecules into the apoplast and/or cytoplasm to manipulate host cells to develop a permanent feeding site [9,10]. Feeding mechanisms differ among PPNs; they are, therefore, a useful tool to group PPN species. Broadly speaking, PPNs are divided into two main categories based on feeding mechanism: endoparasitic and ectoparasitic. Ectoparasitic nematodes occupy clades 1 (Trichodoridae) and 2 (Longidoridae) in the Nematoda lineage; they feed on cortical root cells from outside the root. Clade 2 ectoparasitic nematodes of the genera *Xiphinema* and *Longidorus* utilize an odontostyle (as opposed to the stomatostylet, a feature common to clades 10 and 12 or onchiostyle in clade 1 PPN) to induce cell enlargement. Although the feeding apparatus used by *Xiphinema* and *Longidorus* is the same, the terminal plant cell feeding sites formed after this intimate biotrophic association are slightly different, with those formed by *Xiphinema* inducing plant cell karyokinesis and those formed by *Longidorus* remaining mononucleate [5]. In contrast to ectoparasitic nematodes, endoparasitic nematodes penetrate and feed within plant roots and have been a tremendous burden on global agricultural production, especially in developing areas like Sub-Saharan Africa, where the resources to diagnose and combat them are limited [11]. Endoparasitic nematodes can be further divided into two sub-categories: migratory and sedentary. Sedentary endoparasitic nematodes, such as the root-knot and the cyst nematodes, form specialized feeding sites, which act as nutritional sinks for the developing nematode. Migratory endoparasitic nematodes such as *Scutellonema bradys* (Steiner and LeHew, 1933) Andrassy, 1958, are a tremendous agricultural and economic burden on yam (*Dioscorea* spp.), a crop that has been a major source of income and an important part of the diet in the western regions of Africa [12,13]. A massive amount of genomic and transcriptomic information has been obtained about many migratory endoparasitic nematodes primarily through application of next-generation sequencing (NGS) technologies [14,15,16,17,18,19,20]. Analysis of this information has opened a novel gateway for researchers worldwide to formulate evidence-based conclusions regarding the biology, phylogenetic relationships, and parasitism mechanisms of these nematodes. In this review, we focus only on some of the economically important migratory endoparasitic PPNs from clades 10 and 12. For in-depth analysis of the effectors and processes targeted by sedentary endoparasites of clade 12, the authors suggest several other reviews [10,21,22].

## 2. The Biology of Migratory Endoparasitic Nematodes

A survey of PPN researchers worldwide ranked the top 10 economically significant and scientifically relevant PPNs [23]. Not surprisingly, migratory endoparasitic nematodes, such as root lesion nematodes (*Pratylenchus* spp.), burrowing nematode (*Radopholus similis* (Cobb, 1893) Thorne, 1949), stem or stem and bulb nematode (*Ditylenchus dipsaci* (Kuhn, 1857) Filipjev, 1936) and pine wood nematode (*Bursaphelenchus xylophilus* (Steiner and Buhrer, 1934) Nickle, 1970) occupy positions 3 to 6 in this list, following sedentary root-knot (*Meloidogyne* spp.) and cyst (*Heterodera and Globodera* spp.) nematode species [23]. With the exception of *B. xylophilus*, in clade 10, both migratory and sedentary endoparasitic nematodes are clustered together in clade 12, indicating the possibility that the evolution of *B. xylophilus* into a plant parasite is a recent and convergent one [23]. *B. xylophilus* is also an exception when compared to the other migratory nematodes in the top ten list, in that, it is vectored by insects—specifically adult *Monochamus* (Cerambycidae) beetles (principal vectors), ovipositing on host pine trees [24]. *B. xylophilus* is a facultative plant-parasite, as it feeds on fungi (fungal mycelial mats) as well as xylem parenchyma of live trees. This feature is unique to *Bursaphelenchus* when compared to species of *Pratylenchus*, *Ditylenchus*, and *Radopholus* and most plant-parasitic nematodes, as they are obligate plant-parasites. However, in yet another exception, a newly discovered species of *Bursaphelenchus* named *B. sycophilus,* does not grow on fungal mycelial mats of *Botrytis cinerea* Pers or possess morphological characteristics like the fungal feeding species in this genus, making this nematode an obligate plant-parasite [25].

In the migratory endoparasitic nematodes, the genus *Pratylenchus*, which comprises of ~70 species, ranks third after sedentary root-knot and cyst nematodes, as an economically devastating pest of numerous agriculturally important crops and fruits [26]. An important feature that aids this pathogen in colonizing such a broad host range is the ability for different species to thrive in tropical and temperate climactic conditions. On the contrary, the other three migratory endoparasitic nematode genera, namely *R. similis* (distributed in tropical and temperate greenhouse conditions), *D. dipsaci* (worldwide specifically temperate regions), and *B. xylophilus* (the northern hemisphere, which is home to its vector, *Monochamus* beetles) have a comparatively narrower geographic distribution [27]. Different survival strategies are employed by these nematodes to survive or overcome harsh climactic conditions. For instance, *D. dipsaci* has been shown to survive for more than 20 years by entering into long-term anhydrobiosis [28]. Another migratory PPN, *B. xylophilus*, has been shown to overwinter in both living and dead tissues of coniferous trees, allowing it to endure long, harsh winters [29]. However, the molecular mechanisms underpinning cold tolerance in PPNs are multipartite and poorly understood at present [30]. Homologs of dauer genes have also been found in the genomic and transcriptome analysis of the burrowing nematode *R. similis*; however, since *R. similis* has not been shown to form dauers, the roles of these genes remain unclear [14,19]. There exist six distinct stages: eggs, four juvenile stages (J1–J4), and adults (female and rarely male) in the typical lifecycle of a PPN. The first molt, J1, occurs in the egg. Following this, hatching of the infective juvenile stage, J2, takes place. In most migratory endoparasitic nematodes, all stages (J2–J4 and adult females) possess the ability to infect host cells. Reproductive strategies employed by the different migratory endoparasitic nematode species described above are also oddly similar. Most of the species are dioecious; however, in the absence of males, alternate strategies are pursued, such as hermaphroditism in case of *R. similis* and parthenogenesis in case of certain lesion nematodes [23].

## 3. Feeding Strategies Employed by Migratory Endoparasitic Nematodes

Sedentary endoparasitic PPNs form multinucleate hypertrophied, permanent feeding sites, referred to as syncytium for cyst nematodes and giant cells for root-knot nematodes, which serve as a source of nutrition for the nematode. In contrast, for the migratory endoparasitic nematodes, the feeding mechanisms are fairly straightforward and comparatively less complex. Of these, the penetration and feeding mechanisms of the root-lesion nematode *P. penetrans* (Cobb, 1917) Filipjev and Schuurmans-Stekhoven, 1941, have been extensively studied [31,32,33,34,35]. *Pratylenchus penetrans* has been shown to feed ectoparasitically and endoparasitically [36]. Its endoparasitic feeding behavior can be classified into three distinct stages, namely root surface probing, root penetration, and infection. In the first stage, nematodes migrate primarily to the root hair region and sometimes closer the root tip, around the zone of elongation [33]. Once there, they ectoparasitically probe local epidermal cells and initiate stylet thrusting at an intensity probably proportional to the cell wall structure and thickness [35]. In the second stage, penetration, the regions next to the root intercellular walls are punctured with the stylet accompanied by a slight pressure from the labial region to allow the nematode to enter and create a gateway for subsequent nematodes to enter the root. Intense stylet thrusting continues during this stage as well. Following penetration and entry, the third stage, infection, is initiated. This stage is divided into two sub-stages, brief and extensive feeding. At the beginning of brief feeding, a small salivation zone is present surrounding the stylet tip when inserted into a host cell. The period of brief feeding differs between different nematode developmental stages with juvenile stages consuming less time (approximately 5 minutes) and the adult stages consuming more (approximately 10 minutes). Cortical cell response to the nematode’s brief feeding period includes cytoplasmic streaming and rarely cell death, but nematode migration following these brief feeding periods induces cell death along the migration path. Additionally, preferential feeding behavior is also seen, with J2 and J3 life stages feeding on the root hair and the higher developmental stages (J4 and adults) feeding on the cortical cells [37]. During extended feeding periods, a relatively prominent salivation zone is formed, following which several cell-wall modifying enzymes (CWME) and effectors are secreted into the host cell prior to ingestions. 

Over many years, numerous CWMEs, such as cellulases, pectate lyases, xylanases and arabinases, have been discovered in migratory and sedentary endoparasitic phytonematodes [38,39,40,41,42,43]. Of these, cellulases have been of particular interest to understand the underlying mechanism of host-parasitism and the role of horizontal gene transfer in the origin of parasitism in phytonematodes. Cellulases breakdown cellulose, the major structural component of plant cell walls. Cellulases of root-knot and cyst nematodes have been extensively studied since they are involved in the preliminary interaction with host tissues. The cellulases secreted by sedentary endoparasitic nematodes during the mechanical root puncturing and migration activity have been shown to be homologous to bacterial cellulases, suggesting a possible ancient horizontal gene transfer (HGT) event between bacteria and nematodes with plant parasitism. Molecular characterization studies have confirmed the presence of four cellulases belonging to the GHF5 family (mainly bacterial cellulases) in the burrowing nematode, *R. similis* [39]. Of these, three showed relatively lower expression in males, most likely since males are non-feeding. In another migratory phytonematode, *B. xylophilus*, three cellulases belonging to the GHF45 family have been discovered [41]. GHF45 cellulases are secreted by many microbes including bacteria and fungi as well as some animals; however, the *B. xylophilus* cellulases have been shown to be of fungal origin as opposed to bacterial origin (and *B. xylophilus* feeds upon and lives in close association with fungi). In-situ hybridization studies have confirmed the expression of nematode cellulases specifically within the esophageal gland secretory cells of these parasites, corroborating the hypothesis that these proteins are secreted during the initial phases of parasitism (penetration and migration). However, parasitism proteins involved in disparate stages of host interaction can also be released by other tissues such as the amphids and hypodermis [10]. Cellulases have also been characterized in the molecular and transcriptomic studies of the lesion nematode *P. penetrans, P. thornei* (Sher and Allen)*, P. vulnus* (Allen and Jensen, 1951), and *P. zeae* Graham, 1951 [15,16,37,44,45]. The *P. penetrans* GHF5 cellulases have shown similarity to those in cyst and root-knot nematodes, enforcing a previous finding that some of the early members of the Pratylenchidae family could be gene donors to the root-knot and cyst nematodes [16,43]. Additionally, due to their expression in all migratory endoparasitic nematode life stages, cellulases, specifically β-1,4-endoglucanases, have been utilized to design diagnostic PCR markers for classification of different *Pratylenchus* species from soil and root samples [46].

In addition to cellulases, another secretory protein group extensively studied in PPNs are pectate lyases. Pectate lyases are involved in the breakdown of pectin, an essential component of the plant cell-wall involved in supporting the cellulose and hemicellulose fiber molecules within and between plant cell-walls [47]. Pectate lyases, specifically PL3, have been cloned and reported in numerous sedentary endoparasitic nematodes such as *Meloidogyne, Globodera*, and *Heterodera* [48,49,50,51,52]. In migratory phytonematodes, PL3 has been discovered in the genomes and transcriptomes of *R. similis* [14], *P. coffeae* (Campos and Villain, 2005), *P. penetrans* [16,17], *D. destructor* Thorne, 1945 [53], and *B*. *xylophilus* [40]. These PL3s are involved in softening the plant cell wall, thereby allowing the nematode to migrate through the intercellular spaces. In sedentary endoparasitic nematodes, close homologs of these pectate lyases secreted from the subventral esophageal glands have been shown to be of a bacterial and fungal origin [54]. Furthermore, RNAi knockdown studies of these pectate lyases in the sedentary endoparasitic phytonematode, *H. schachtii* Schmidt, 1871, culminated in fewer nematodes per root tissue sample of *Arabidopsis thaliana* (L.) Heynhold, 1976 plants, implicating the need for these enzymes in parasitism [55]. CWMEs such as arabinases, xylanases, and polygalactouranases have also been found during genomic and transcriptomic analyses of multiple parasitic phytonematodes [2,38,47,56]. However, extensive molecular characterization studies elucidating the interplay between different CWMEs during parasitism in migratory PPNs have not been performed, thereby leaving a significant gap in the knowledge necessary to understand the processes taking place during the course of infection by a migratory PPN. 

## 4. Parasitism Gene Repertoire and Effectors of Migratory Endoparasitic Nematodes

Low-cost and resource-efficient NGS technologies have opened gateways for researchers to analyze the arsenal of putative effectors secreted by PPNs. The broad definition of effectors proposed by Hogenhout et al., “all pathogen proteins and small molecules that alter host-cell structure and function”, encompasses the spectrum of effectors secreted by PPNs [57]. PPN effectors are generally expressed and secreted by esophageal gland cells, specifically one dorsal and two subventral esophageal gland cells, through the stylet. The role of these esophageal gland cells in sedentary endoparasitic PPN is generally regulated; the subventral gland cells are metabolically active during the pre-parasitic and early infective stages, and the dorsal gland cells are active during a more permanent association with the host tissues [10,37]. An overview of some of the key parasitism genes discovered in economically important migratory PPNs since 2013 is provided in Table 1.

### 4.1. Pratylenchus spp.

In *P. penetrans*, numerous effectors have been identified and their putative role in parasitism has been deciphered [16,45]. Some of the significant effectors discovered in the *P. penetrans* transcriptome include catalases (with N-terminal signal peptide) and glutathione peroxidase that play role in shielding the nematode against host induced reactive oxygen species (ROS) molecules. Moreover, notable effectors identified in the transcriptome dataset include the venom allergen-like proteins (VAPs) that have also been identified in *B. xylophilus* and have been hypothesized to be involved in movement within the host plant [67]. Furthermore, effectors such as transthyretin-like proteins (TTLs) and fatty-acid and retinol-binding proteins (FARs) were also identified in the *P. penetrans* transcriptome dataset [45]. TTLs have been implicated to play an important role in the nervous system of *R. similis* and FARs from the cyst, and root-knot nematodes have been shown to bind precursor molecules involved in the defense related jasmonic acid signaling pathway [68,69,70]. During the comparative studies of root-lesion nematodes, several genes coding for PPN effectors involved in initiating a feeding site in host tissues were noted to be absent [15,16,17].

Transcriptome analysis of another member of this genus, *P. coffeae*, revealed several proteins homologous to effectors involved in parasitism [17]. Notable amongst them are the genes coding for chorismate mutase, glutathione peroxidase, glutathione-S-transferase peroxiredoxin, TTLs, and VAPs. A noteworthy finding of this study was the identification of proteins homologous to RBP-1, a cyst nematode secretory protein with an SP1a and ryanodine receptor domain (SPRYSEC). SPRYSECs have been shown to both suppress and elicit immune response in plants and within PPNs; these proteins have been identified only in the sedentary cyst nematodes [71]. The authors identified four putative SPRYSEC proteins in *P. coffeae* with a significantly high homology to the cyst nematode SPRYSECs [17]. In addition, the authors identified other SPRY domain containing proteins in *P. coffeae* as well. However, these do not show any similarity with the cyst nematode SPRYSEC proteins. A putative gene coding for an arabinogalactan galactosidase, a protein found only in the cyst nematodes, was also identified in *P. coffeae*. Additionally, many genes involved in cell-wall modification, such as β-1,4-endo-glucanase, pectate lyase, xylanase, and GH16 (β-1,3-endoglucanases), were uncovered during the transcriptome analysis of *P. coffeae* [17]. However, no GH16s were identified in the transcriptome of *P. penetrans*, indicating the evolution of a distinct effector set in *P. penetrans* as a result of host/niche specialization [16].

Transcriptome analysis of another root-lesion nematode, *P. zeae*, an important pest of high-value crops such as sugarcane (*Saccharum officinarum* L.) and sorghum (*Sorghum bicolor* (L.) Moench), also revealed a similar trend in CWME occurrence [15]. *Pratylenchus zeae* possesses a similar suite of effectors when compared with close relatives, *P. penetrans* and *P. coffeae*. Notable in the transcriptome were the genes involved in combating host-derived oxidative stresses, such as glutathione S-transferase, peroxiredoxin, thioredoxin, glutathione peroxidase, and superoxide dismutase [15]. Additionally, genes identified in the transcriptome dataset of *P. coffeae* such as the cyst nematode secreted SPRYSEC proteins were also identified in *P. zeae*. Analysis of the putative secretome of *P. zeae* revealed several sequences involved in a variety of functions such as stress response, energy metabolism, protein digestion, host defense evasion, and plant cell wall modification [15]. Notably, the authors found expression of a gene with a SPRY domain localized within the nematode esophageal gland cells, implicating it in the parasitism process. The authors also noted that several genes involved in feeding site formation induced by sedentary nematodes such as C-terminally encoded peptides (CEP), CLE-like peptides, 16D10, and 7E12 were missing from the transcriptome analysis of *P. zeae*. Another important absence is of a gene coding for a putative chorismate mutase in *P. zeae*, which is present in the transcriptomes of *P. coffeae* and *P. thornei*. 

### 4.2. Radopholus similis

Of the 30 described species of *Radopholus*, *R. similis* is the only major burrowing nematode species considered significant worldwide [72,73]. The transcriptome of *R. similis* has been sequenced twice [14,18]. Genes identified in *R. similis* include those involved in plant cell wall modification such as β-1,4-endoglucanase, pectate lyase, endoxylanase, arabinase, and expansins and in parasitism, genes were identified in few root-lesion nematodes such as SXP/RAL-2, FAR and chorismate mutase. Proteinase-coding genes such as serine carboxypeptidase, calreticulin, and cathepsin that have been linked to different aspects of plant parasitism were also identified in *R. similis* [58,59,61,69]. Similar to few root-lesion nematodes, homologues of sequences involved in sedentary endoparasitism such as CLE, CEP, 16D10, and 7E12 were absent in *R. similis*. Several gene sequences coding for SPRY domains were identified in the genome analysis. However, no N-terminal signal peptides were found in these proteins. Another notable finding in the *R. similis* genome, as well as transcriptome analysis, is the presence of several genes involved in the dauer pathway. A study by Chabrier et al. demonstrated after 180 days under no host conditions, *R. similis* males increased in number (21.7%) compared to females (9.8%), indicating a possibility of higher expression of dauer genes in males than females (or lower survival of females during unfavorable conditions, and hence a lower expression of dauer genes) [74].

The recent genome analysis of *R. similis* has also shed more light into the relationship of this migratory nematode with the sedentary cyst nematodes. A recent robust phylogenetic analysis of the small subunit ribosomal DNA (SSU rDNA) by Holterman et al. revealed five lineages leading to sedentary endoparasitism. Among these, the subfamilies Pratylenchinae and Hirschmanniellinae were hypothesized as ancestors of the sedentary root-knot nematodes. Additionally, a common ancestral link between the semi-endoparasitic nematode, *Rotylenchulus reniformis* Linford and Oliveira, 1940 and the endoparasitic cyst nematodes has also been suggested by rDNA analysis as well as the effector analysis [75,76]. In the same phylogenetic analysis, it can be noted that *R. similis* and the members of the genus *Heterodera*, *Globodera*, and *Rotylenchulus* share a common ancestor. However, although it is relatively phylogenetically close, *R. similis* shares no overlap of key effectors such as CLE and SPRYSEC with the sedentary cyst nematodes. Holterman et al. also demonstrated the close association between members of the *Radopholus* genus, specifically *R. bridgei* and *R. similis* with *Hoplolaimus femina*. *Hoplolaimus* is comprised of phytonematodes with a wide range of lifestyle (ecto, endo, and semi-endoparasites) [77]. However, limited information is available regarding the molecular interactions governing parasitism for this nematode (*Hoplolaimus* genus) or the similarity/differences of effector repertoire between the other two *Radopholus* species (*R. bridgei* Siddiqi and Hahn, 1995 and *R. similis*).

### 4.3. Ditylenchus spp.

Expressed sequence tag (EST) analyses has been used previously to identify several genes involved in parasitism in the root-knot and cyst forming nematodes [78,79]. EST analysis of the potato rot nematode, *D. destructor*, revealed homologs of several important effectors such as VAP and calreticulin, which play vital roles in host defense induction. Moreover, effectors involved in circumventing host defense in cyst nematodes such as SEC-2 proteins and numerous cell wall modifying enzymes, such as pectate lyase, cellulase, and expansin, were also identified in *D. destructor* [63,70]. Additionally, 14-3-3b a secretory protein identified in the gland cell of *M. incognita* (Kofoid and White, 1919) Chitwood, 1949 and implicated in playing an essential role in signal transduction pathways, has also been identified in the EST dataset of *D. destructor* [63,80]. Since *D. destructor* feeds on fungi as well, it is unsurprising that proteins involved in fungal cell wall degradation, such as chitinases and GH16 (1-3(4))-beta-glucanase genes, were identified in the EST dataset of this nematode.

EST analysis of the peanut pod nematode, *D. africanus* Wendt, Swart, Vrain, and Webster, 1995, identified a similar suite of expressed parasitism genes [81]. Homologs of key genes participating in anhydrobiosis, such as the late embryogenesis abundant protein (LEA), were also identified in *D. africanus*. This is on par with certain members of the genus *Ditylenchus*, such as *D. dipsaci*, which have been shown to be capable of anhydrobiosis [82]. Genes involved in providing structural integrity to the nematode cell membrane, such as fatty acid desaturase and stomatin, were also identified in the EST dataset [81].

### 4.4. Bursaphelenchus spp.

Analysis of over 13,000 and 3000 ESTs from *B. xylophilus* and *B. mucronatus* (Mamiya and Enda, 1979), respectively, revealed several genes involved in parasitism [83]. In addition to the conventional parasitism genes, genes such as chitinase, expressed in the esophageal gland cells of the cyst nematode, have also been found in *B. xylophilus* [83,84]. Presence of this and other chitin degrading enzymes, like GH16, are necessary because *Bursaphelenchus* feeds on fungi (cell wall made of chitin) and is vectored by *Monochamus* insects (insect cuticle is made of chitin). Additionally, with *D. africanus,* the authors found several dauer genes such as LEA homologs as well as 18 *Caenorhabditis elegans* (Maupas, 1899) dauer-formation (*daf)* homologs. Identification and characterization of parasitism genes has led to the discovery of a multilayered enzymatic detoxification strategy in *B. xylophilus*, with detoxification enzymes such as glutathione S-transferase being secreted as the first line of response, and other parasitism effectors such as VAP being secreted later [66]. Detoxification enzymes such as glutathione S-transferase have been shown to play significant roles towards plant parasitism in root-knot nematodes [66,85]. Recently, the effector gene, *BxSapB1*, has been identified in *B. xylophilus* [66]. *BxSapB1* possesses a functional signal peptide and has been demonstrated to contribute towards host cell death and increased virulence of *B. xylophilus* [64]. 

## 5. Management of Migratory PPNs

A variety of management strategies has been employed for different types of PPN due to their ability to parasitize a wide range of hosts. Migratory PPNs such as those belonging to the genera *Radopholus*, *Ditylenchus*, and *Pratylenchus* can be easily spread by contaminated vegetative plant parts and timber (in the case of *B. xylophilus*) [86]. Integrating multiple management strategies is essential; however, the specific strategy should depend upon accurate diagnosis of the PPN species, growing conditions, available resources, and economic feasibility, which vary among cropping systems and in developing vs. developed countries.

### 5.1. Cultural Practices

Cultural practices such as crop rotation with a non-host crop or instituting a period of fallow (including elimination of weeds), can reduce nematode populations. Crop rotation is less effective as a control strategy, however, due to the polyphagous nature of some PPNs. For instance, rotation with crops such as cassava (*Manihot esculenta)* and sweetpotato (*Ipomoea batatas*) has been useful in bringing down *R. similis* populations [87], but sweet potato is a susceptible host for other types of PPN such as *M. enterolobii* Yang and Eisenback, 1981 and *Rotylenchulus reniformis*. However, in the case of *P. zeae*, effective control has been achieved by rotating rice with leguminous beans such as black gram (*Vigna Mungo* (L.) Hepper) and mung bean (*Vigna radiata* (L.) R. Wilczek) [88]. Control of another lesion nematode, *P. thornei* in wheat (*Triticum aestivum*), was also achieved in Mexico by rotating with crops such as cotton (*Gossypium* spp.), corn (*Zea mays*), and soybean (*Glycine max*) for two successive years [89]. The use of certified nematode-free starting material such as seed potato (*Solanum tuberosum*) or banana (*Musa* spp.) corm has been helpful in reducing nematode populations of *D. dipsaci* and *R. similis*, respectively [90,91,92]. Use of clean starting material has also been effective in reducing populations of the yam nematode *S. bradys*, the causative agent of dry rot disease in yams. Utilizing hot water treatment for disinfecting starting plant material has been useful for managing populations of *D. dipsaci, P. vulnus*, and *R. similis* [90,91,92]. 

Cover crops are an indispensable unit in an agricultural ecosystem since they provides a host of benefits to the soil such as increased nutrients, reduced pest populations, as well as an increase in beneficial soil microbes. Use of marigold (*Tagetes* spp.), which has been shown to produce thiophene α terthienyl (a nematicidal compound), as a cover crop has provided effective control against *P. penetrans* [93,94,95,96]. Sunn hemp (*Crotalaria juncea* L.), a leguminous cover crop, has also shown significant potential in reducing populations of several sedentary nematodes of the *Meloidogyne* and *Heterodera* genera as well as migratory nematodes such as the sting nematode (*Belonolaimus longicaudatus* Rau, 1958) and stubby root nematodes (*Paratrichodorus* and *Trichodorus* spp.) [97,98]. Moreover, recent studies with sunn hemp, pigeon pea (*Cajanus cajan*), and *Gliricidia sepium* have delivered promising results in managing the endoparasitic yam nematode, *S. bradys* [99]. However, a recent trial with sunn hemp and pigeon pea showed no nematode suppression activity on the migratory nematode *R. similis* [100]. In addition, cover crops such as mustard (*Brassica* and *Sinapis* spp.) contain a wide variety of PPN antagonistic compounds such as isothiocyanates and degradation products of glucosinolates, which have provided moderate nematode suppression effects [101]. For a detailed review of phytochemicals for nematode control, the authors recommend Chitwood [102].

### 5.2. Resistance as a Tool for Nematode Control

Due to the growing environmental and human health concerns caused by the use of toxic nematicides and the associated regulations surrounding their use, incorporating natural resistance (R) genes into desirable crop cultivars has been a successful strategy to provide crop protection against PPNs [103]. The first cloned naturally occurring R gene that provided resistance against a PPN was the Hs1^pro1^ gene from sugar beet (*Beta vulgaris*) [104,105]. This gene confers resistance to the sedentary cyst-forming nematode, *H. schachtii* [104]. Since then, many nematode R genes have been cloned and identified including *Mi-1, Hero A*, *Gpa2, Gro1-4*, *Rhg1*, and *Rhg4*; however, these genes confer resistance only to sedentary PPNs [106,107,108,109,110,111,112].

With regards to migratory nematodes, Atkinson et al. (2004) [113] demonstrated the efficacy of the first transgenic bananas expressing rice cystatins which provided significant resistance (around 70% ± 10%) against *R. similis.* Transgenic *Nicotiana benthamiana* plants expressing the cysteine proteinase cathepsin S also demonstrated enhanced resistance to *R. similis* [114]. Musa varieties such as the Yangambi Km5 and Pisang Jari Buaya also provide some degree of resistance against *R. similis*, primarily through mechanisms involving phenol accumulation and lignification, respectively, at the nematode infection site [115,116]. Furthermore, evidence of a mutualistic Fusarium endophyte (*Fusarium oxysporum* isolate A1 and *Fusarium* cf. *diversisporum*) inducing systemic resistance against *R. similis* in bananas has also been demonstrated [117]. Another instance of resistance against a migratory PPN is the bread wheat line Gatcher selection 50a, which provides partial resistance against the root lesion nematode *P. thornei* [118]. Partial resistance to *P. thornei* has also been identified in other varieties of wheat such as "CPI133872", grown in multiple regions in Australia, where *P. thornei* is an extremely damaging pathogen [119,120,121]. Although, several trials on potato varieties have been conducted to identify sources of resistance or tolerance (‘tolerance" as defined by Mwaura et al.) against nematodes in the *Ditylenchus* genus, few varieties are commercially available [122]. In a recent study based on the relative susceptibility score, the potato variety "Spunta" was classified as resistant against *D. destructor* and *D. dipsaci* under greenhouse conditions [122]. However, these results have not been confirmed under field conditions. 

### 5.3. Nematicides

Due to the deleterious effects of certain nematicides/pesticides on the environment and non-target organisms, severe regulatory restrictions including the ban of important nematicides have been established. One such broad spectrum pesticide, methyl bromide, which is also a popular fumigant nematicide, was phased out of agricultural use in the United States in 2005 due to its atmospheric ozone depletion properties [123,124,125]. Currently there exists a huge gap in the number of effective and economical nematicide products that are available to growers. Some of the important fumigant nematicides currently approved for use on high-value crops and ornamentals in the United States include metam sodium (Vapam^®^), metam potassium (Metam CLR™), chloropicrin (Metapicrin), and 1,3-dichloropropene (Telone^®^) [125]. An alternative to methyl bromide with regards to the lowest per-unit cost is the registered fumigant chloropicrin, which has been used in combination with the fumigant nematicide 1,3-D [126]. This combination treatment has provided effective control against soilborne pathogens in a broad variety of crops such as almonds (*Prunus dulcis,* syn. *Prunus amygdalus)*, sweetpotatoes, strawberries (*Fragaria anannasa*), grapes (*Vitis vinifera*), and carrots (*Daucus carota* subsp. *sativus*), but limited control of weeds and high input costs concern many growers. Additionally, application of 1,3-D has been restricted within California townships, with a ban of 1,3-D in the month of December due to air quality concerns [127,128]. Another example is the non-fumigant nematicide, fenamiphos (Nemacur^®^). Fenamiphos has been utilized by *Anthurium* growers in Hawaii as a post-plant application for managing *R. similis* and other PPNs [129]. However, with a phase-out of this chemical in the U.S., growers are now integrating cleaner management practices such as using micropropogated *Anthurium* plantlets and disinfested starting materials [130]. An alternative microbial nematicide, DiTera^®^ (nematicide synthesized from the fungus *Myrothecium verrucaria*) has also been recommended as an effective low-risk nematicide for reducing *R. similis* populations in *Anthurium* [130]. In a recent study by Zouhar et al., treatment of garlic (*Allium sativum)* cloves with the fumigant hydrogen cyanide at a concentration of 20g/m^3^ caused significant increase in *D. dipsaci* mortality [131].

Post-plant application of the systemic nonfumigant nematicide oxamyl (Vydate^®^) has provided effective control against the root-lesion nematode, *P. penetrans*, in raspberries (*Rubus idaeus)* during field trials in Washington [132]. Another nonfumigant nematicide that has been registered in several countries for crop protection against multiple nematodes, predominantly the root-lesion, potato rot, and pine wood nematode, is the contact nematicide fosthiazate (Nemathorin^®^) [133]. Migratory PPNs such as root-lesion nematodes generally occur as a complex with other plant-pathogens such as *Fusarium* spp. [134]. Trials with a combination of abamectin–fungicide coated seeds reduced root infection by *P. penetrans* in maize [135]. Seed treatments are also an important form of control for the stem and bulb nematode *D. dipsaci* [131]. Novel seed treatment strategies such as electrospinning of agrichemicals has also recently shown promise to manage plant pathogens under laboratory conditions [136]. Essential oils and volatiles derived from several families of Portuguese aromatic flora have also shown effective nematicidal properties against the pine-wilt nematode *B. xylophilus* [137]. Although, several significant nematicides have been phased out and severe regulations have been imposed on some of the remaining ones, a management strategy combining multiple control practices should be practiced for effective nematode control.

## 6. Conclusions

Migratory PPNs are distributed across multiple clades in the phylum Nematoda. Although there exist similarities in the biology and life cycle of several migratory PPNs, huge contrasts and divergences are seen with respect to the anatomical adjustments made to survive in the absence of a viable host. Parallel evolution of feeding enzymes that allow successful feeding in the host cells and on other microbes such as fungi is a trait that has undergone divergence several times in the course of evolution of migratory PPNs in the Nematoda lineage. By coupling spatio-temporal NGS approaches with molecular/functional characterization studies, insights into some of the key effectors and their targets can be gained, which shed light on the multifaceted interaction of a PPN with its host. Migratory PPNs, like their sedentary counterparts, have been distributed across the globe, probably as a result of increased international trade. A multipronged control strategy that depends less on chemical means and integrates factors such as the geographical location, nematode species and host range, plant resistance, as well as sound agricultural practices should be considered during the decision-making process for managing any plant-parasitic nematode.

## Figures and Tables

**Table 1:** List of some of the significant parasitism genes in the four key migratory plant-parasitic nematode (PPN) genera published since 2013.

Gene Name/Cluster ID	Species	Function/Annotation	Reference
*Pratylenchus* spp.			
*Ppen12895_c0_seq1 (FAR)*	*P. penetrans*	Fatty-acid metabolism	[45]
*Ppen12103_c0_seq1* (SXP-RAL2)	*P. penetrans*	Function in parasitism: unclear	[45]
*Vap-1*	*P. zeae*	Host defense suppression	[15]
*Sec-2*	*P. zeae*	Overcoming host defense	[15]
*Radopholus similis*			
*Rs-scp-1*	*R. similis*	Development, invasion, and pathogenesis in some PPN	[58]
*Rs-cps*	*R. similis*	Embryonic development invasion and pathogenesis	[59]
*Rs-cb-1*	*R. similis*	Reproduction and invasion	[60]
*Rs-crt*	*R. similis*	Reproduction and pathogenicity	[61]
*Rs-far-1*	*R. similis*	Development, reproduction, infection, and disruption of plant defense	[62]
*Ditylenchus* spp.			
DD03093(VAP-1)	*D. destructor*	Host defense suppression	[63]
DDC03397(VAP-2)	*D. destructor*	Host defense suppression	[63]
DD03835 (Sec-2)	*D. destructor*	Overcome host defense	[63]
*Bursaphelenchus* spp.			
BxSapB1	*B. xylophilus*	Contributes to virulence and cell death	[64]
1-3-endoglucanase	*B. xylophilus*	Cell-wall degrading enzymes	[65]
Expansin-like protein	*B. xylophilus*	Cell-wall degrading enzymes	[65]
Peroxiredoxin	*B. xylophilus*	Detoxifying enzyme	[65]
Cytochrome-P450	*B. xylophilus*	Detoxifying enzyme	[66]
Glutathione-S-transferase	*B. xylophilus*	Detoxifying enzyme	[66]
